# Direct thrombin inhibitors as alternatives to heparin to preserve lung growth and function in a murine model of compensatory lung growth

**DOI:** 10.1038/s41598-022-25773-3

**Published:** 2022-12-07

**Authors:** Savas T. Tsikis, Thomas I. Hirsch, Scott C. Fligor, Amy Pan, Malachi M. Joiner, Angela Devietro, Paul D. Mitchell, Hiroko Kishikawa, Kathleen M. Gura, Mark Puder

**Affiliations:** 1 Vascular Biology Program, Boston Children’s Hospital, Harvard Medical School, Boston, MA 02115 USA; 2 Department of Surgery, Boston Children’s Hospital, Harvard Medical School, 300 Longwood Ave, Fegan 3, Boston, MA 02115 USA; 3 Institutional Centers for Clinical and Translational Research, Boston Children’s Hospital, Boston, MA 02115 USA; 4 Department of Pharmacy and the Division of Gastroenterology and Nutrition, Boston Children’s Hospital, Harvard Medical School, Boston, MA USA

**Keywords:** Health care, Paediatrics, Paediatric research, Physiology, Respiration

## Abstract

Infants with congenital diaphragmatic hernia (CDH) may require cardiopulmonary bypass and systemic anticoagulation. Expeditious lung growth while on bypass is essential for survival. Previously, we demonstrated that heparin impairs lung growth and function in a murine model of compensatory lung growth (CLG). We investigated the effects of the direct thrombin inhibitors (DTIs) bivalirudin and argatroban. In vitro assays of lung endothelial cell proliferation and apoptosis were performed. C57BL/6 J mice underwent left pneumonectomy and subcutaneous implantation of osmotic pumps. Pumps were pre-loaded with normal saline (control), bivalirudin, argatroban, or heparin and outcomes were assessed on postoperative day 8. Heparin administration inhibited endothelial cell proliferation in vitro and significantly decreased lung volume in vivo, while bivalirudin and argatroban preserved lung growth. These findings correlated with changes in alveolarization on morphometric analysis. Treadmill exercise tolerance testing demonstrated impaired exercise performance in heparinized mice; bivalirudin/argatroban did not affect exercise tolerance. On lung protein analysis, heparin decreased angiogenic signaling which was not impacted by bivalirudin or argatroban. Together, this data supports the use of DTIs as alternatives to heparin for systemic anticoagulation in CDH patients on bypass. Based on this work, clinical studies on the impact of heparin and DTIs on CDH outcomes are warranted.

## Introduction

Congenital diaphragmatic hernia (CDH) occurs as a result of a developmental defect in the diaphragm with a prevalence of 1 to 4 cases per 10,000 live births^1,2^. Mortality in CDH primarily arises from the resulting pulmonary hypoplasia that occurs in both lungs^3,4^. Even following surgical repair, many patients require extended periods of cardiopulmonary bypass through extracorporeal membrane oxygenation (ECMO) and systemic anticoagulation. Expeditious lung growth while on ECMO is necessary to improve the substantial mortality (up to 50%) observed in CDH as longer run times are associated with worse outcomes^5^.

Murine left pneumonectomy induces compensatory lung growth (CLG) in the remaining right lung^6,7^. This serves as an animal model for CDH and other hypoplastic lung diseases given similarities in lung alveolarization and molecular signaling to human lung development^8^. We previously demonstrated that CLG is complete starting on post-operative day (POD) 8 following left pneumonectomy^9^. In this model, exogenous heparin administration resulted in decreased lung volumes, pulmonary function, and inhibition of pro-angiogenic signaling involving the vascular endothelial growth factor (VEGF) pathway^10^. Heparin is the most common choice of anticoagulant utilized to maintain circuit patency in CDH patients requiring ECMO. Therefore, these data suggest that the anticoagulation strategy for these patients requires reconsideration.

Bivalirudin and argatroban are synthetic peptide derivatives that exert their anticoagulant effects as direct thrombin inhibitors (DTIs)^11^. DTIs are increasingly being utilized to maintain circuit patency during cardiopulmonary bypass and ECMO^12,13^. Initial studies from our laboratory suggest that anticoagulation with bivalirudin does not affect CLG^10,14^. This work was limited as dosing was via intermittent intraperitoneal injection rather than continuous anticoagulation, and bivalirudin has a short half-life. Prior studies have demonstrated the successful continuous delivery of argatroban using osmotic pumps in mice^15^. However, no prior study, to our knowledge, has evaluated the dosing required to achieve and maintain continuous systemic anticoagulation in the murine model.

In this study, we first evaluate the effects of bivalirudin, argatroban, and heparin on lung endothelial cells in vitro*.* We then investigate the use of these anticoagulants with osmotic pumps to achieve continuous systemic anticoagulation in mice. Following appropriate dose–response studies, we evaluate their effect on lung growth and function using the murine model of CLG in vivo. Since DTIs have a more specific mechanism of action compared to heparin, we hypothesized that DTI treatment, unlike heparin, would not impact lung growth and function.

## Results

### Heparin inhibited lung endothelial cell proliferation and increased apoptosis

**Figure 1:**
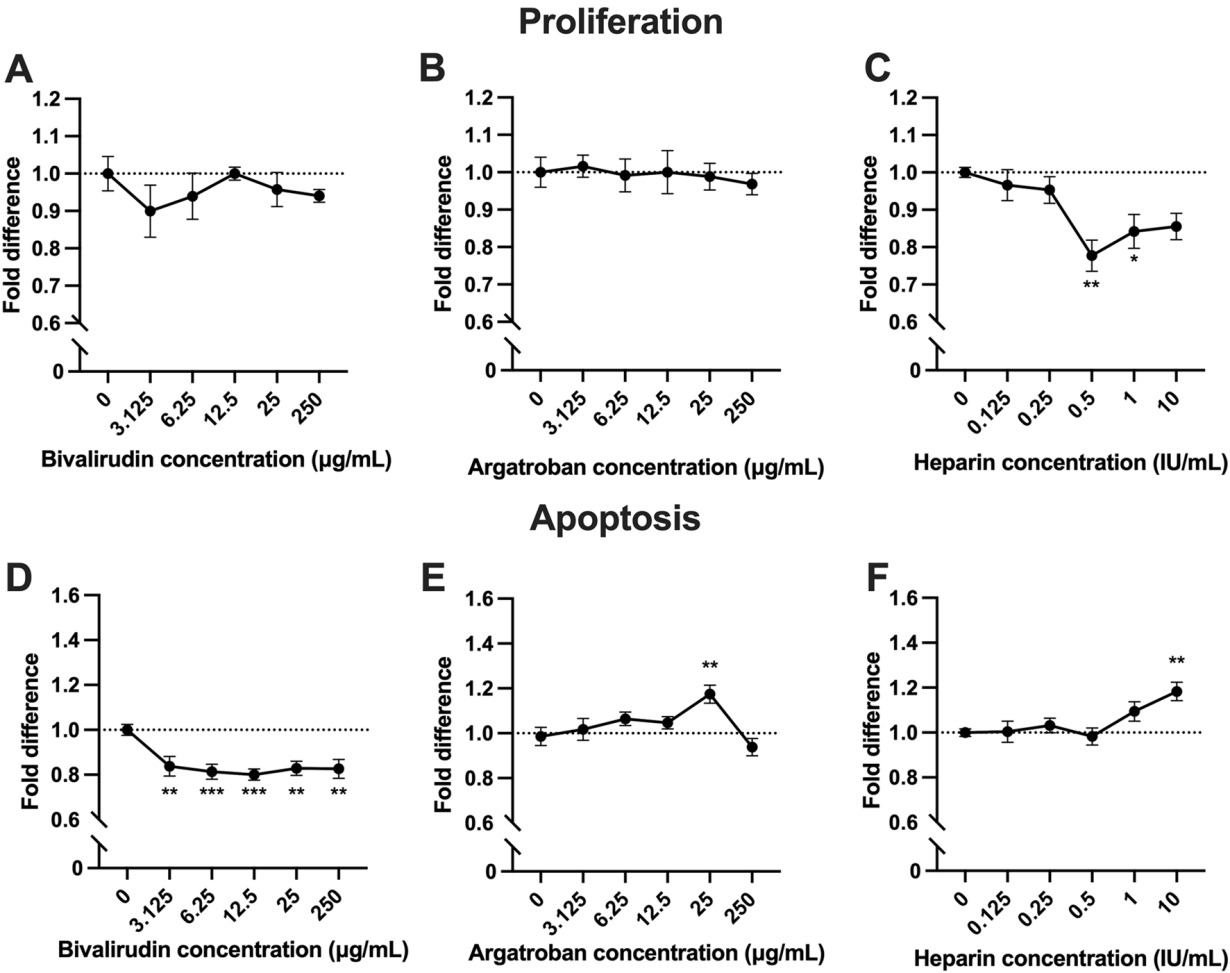
Lung endothelial cell proliferation and apoptosis. Bivalirudin (**A**) and argatroban (**B**) did not affect human lung microvascular endothelial cell (HMVEC-L)-L proliferation at various dosages. Heparin, however, inhibited proliferation of HMVEC-L cells in a dose-dependent manner (**C**). Bivalirudin consistently inhibited apoptosis (**D**). Argatroban increased apoptosis at a single dose (25 μg/mL) while remaining doses of argatroban did not impact apoptosis (**E**). Increasing doses of heparin increased HMVEC-L apoptosis (**F**). Each anticoagulant dose was performed in technical quadruplicate and the experiments were repeated up to 3 times. Comparisons were done using a one-way analysis of variance (ANOVA) with Dunnett’s adjustment for multiple comparisons. Shown are mean ± standard error. *P < 0.05; **P < 0.01; ***P < 0.001.

The effects of bivalirudin, argatroban and heparin on proliferation and apoptosis of human lung microvascular endothelial cells (HMVEC-L) were evaluated. There was no significant effect on HMVEC-L proliferation with increasing dosages of bivalirudin or argatroban (Fig. 1A,B). Heparin significantly inhibited HMVEC-L proliferation (Fig. 1C) at the 0.5 IU/mL (0.78 ± 0.06-fold, *P* = 0.008) and 1 IU/mL dosages (0.84 ± 0.06-fold, *P* = 0.046). The even higher 10 IU/mL heparin dose was also inhibitory but did not reach statistical significance (0.85 ± 0.06-fold, *P* = 0.072).

Interestingly, bivalirudin consistently inhibited HMVEC-L cell apoptosis at all doses (Fig. 1D). Argatroban increased apoptosis at the 25 μg/mL dose (Fig. 1E; 1.17 ± 0.05-fold, *P* = 0.003) but the 250 μg/mL dose did not affect apoptosis (*P* = 0.83). Heparin increased apoptosis in a dose-dependent manner (Fig. 1F), with the 10 IU/mL dose reaching statistical significance (1.18 ± 0.05-fold, *P* = 0.006).

### Continuous administration of bivalirudin and argatroban resulted in therapeutic anticoagulation

Prior to pump implantation in CLG mice, we first evaluated the dosing required to achieve and maintain continuous systemic anticoagulation (Supplementary Fig. S1). Different concentrations and rates of drug delivery were trialed. Increasing drug dosages consistently increased the level of anticoagulation (Supplementary Fig. S1A) as measured by the partial thromboplastin time (PTT). Bivalirudin resulted in supratherapeutic anticoagulation compared to saline-treated controls at the 50 μg/hr dose (105 ± 6.3 vs. 35.4 ± 5.8 s, *P* = 0.03) while argatroban-treated mice consistently achieved supratherapeutic levels at the 50 μg/hr (143 ± 6.7 vs. 35.4 ± 5.8 s, *P* = 0.002) and 100 μg/hr (150 ± 10.5 vs. 35.4 ± 5.8 s, *P* = 0.001) dosages. Heparin contributed to supratherapeutic anticoagulation at the 2.5 IU/hr (194 ± 6.2 vs. 35.4 ± 5.8 s, *P* = 0.0001) and 5 IU/hr (> 200 ± 0 vs. 35.4 ± 5.8 s, *P* < 0.0001) dosages.

The lowest dose of each anticoagulant resulting in supratherapeutic anticoagulation was then selected for longitudinal studies (Supplementary Fig. S1B–D). Both bivalirudin and argatroban maintained their anticoagulation effect assessed at various time-points during the eight day study. Heparin-treated mice were noted to have decreasing PTTs, reaching comparable levels to controls starting on day 4 (Supplementary Fig. S1D). These mice received an additional daily intraperitoneal injection of heparin (0.5 IU/g)^10^ to maintain therapeutic anticoagulation during the remainder of the study. To ensure that heparinized mice were adequately anticoagulated on POD 8 following pneumonectomy, anti-factor Xa levels were compared across the groups at that time point (Supplementary Fig. S1E). Anti-factor Xa activity is used clinically to evaluate the anticoagulation effects of heparin and is a surrogate marker for plasma heparin concentration. The heparin group had significantly increased anti-factor Xa levels compared to controls (0.86 ± 0.20 vs. 0.23 ± 0.017 IU/mL, *P* = 0.001). Given that bivalirudin and argatroban act downstream in the coagulation cascade, these anticoagulants should not increase anti-factor Xa levels. We did not observe any significant change in anti-factor Xa levels in these groups (*P* = 0.98, *P* = 1.00, respectively). The study groups and final anticoagulant dosing regimens utilized in this study are provided in Supplementary Table S1.

### Heparin decreased lung volume while bivalirudin and argatroban did not affect lung growth and function

**Figure 2:**
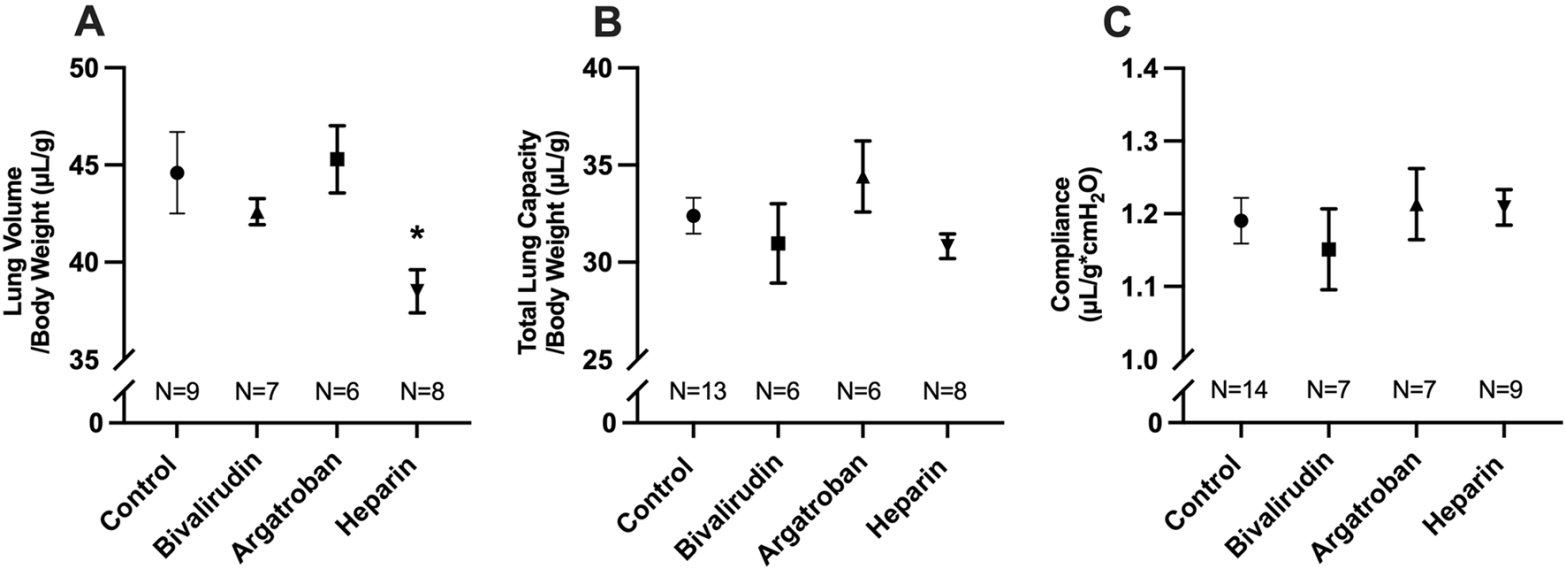
Lung growth and pulmonary function testing. Heparin administration impaired compensatory lung growth, as demonstrated by significantly decreased lung volume compared to controls (**A**). Lung volume was not different in the bivalirudin or argatroban groups. Total lung capacity was lowest for heparinized mice, although there were no significant differences between groups (**B**). Although heparin affected lung growth, there were no differences in pulmonary compliance across all four groups (**C**). Statistical analysis of the experimental groups was done using analysis of variance (ANOVA), with Dunnett’s adjustment for multiple comparisons. TLC and lung volumes were normalized to mouse body weight. Shown are mean ± standard error. *P < 0.05.

Lung growth and pulmonary function testing (PFT) were assessed at POD 8 following left pneumonectomy and pump implantation (Fig. 2). Bivalirudin (42.6 ± 0.7 vs. 44.6 ± 2.1 μL/g, *P* = 0.70) or argatroban (45.3 ± 1.7 vs. 44.6 ± 2.1 μL/g, *P* = 0.98) did not significantly change lung volume. Heparinized mice demonstrated significantly decreased lung volume (Fig. 2A) compared to controls (38.5 ± 1.1 vs. 44.6 ± 2.1 μL/g, *P* = 0.02). There were no significant differences in total lung capacity (TLC) between the four groups (Fig. 2B). Although heparin significantly affected lung growth, there were no differences in pulmonary compliance across the four experimental groups (Fig. 2C).

### Bivalirudin and argatroban, but not heparin, preserved lung alveolarization following compensatory lung growth

**Figure 3:**
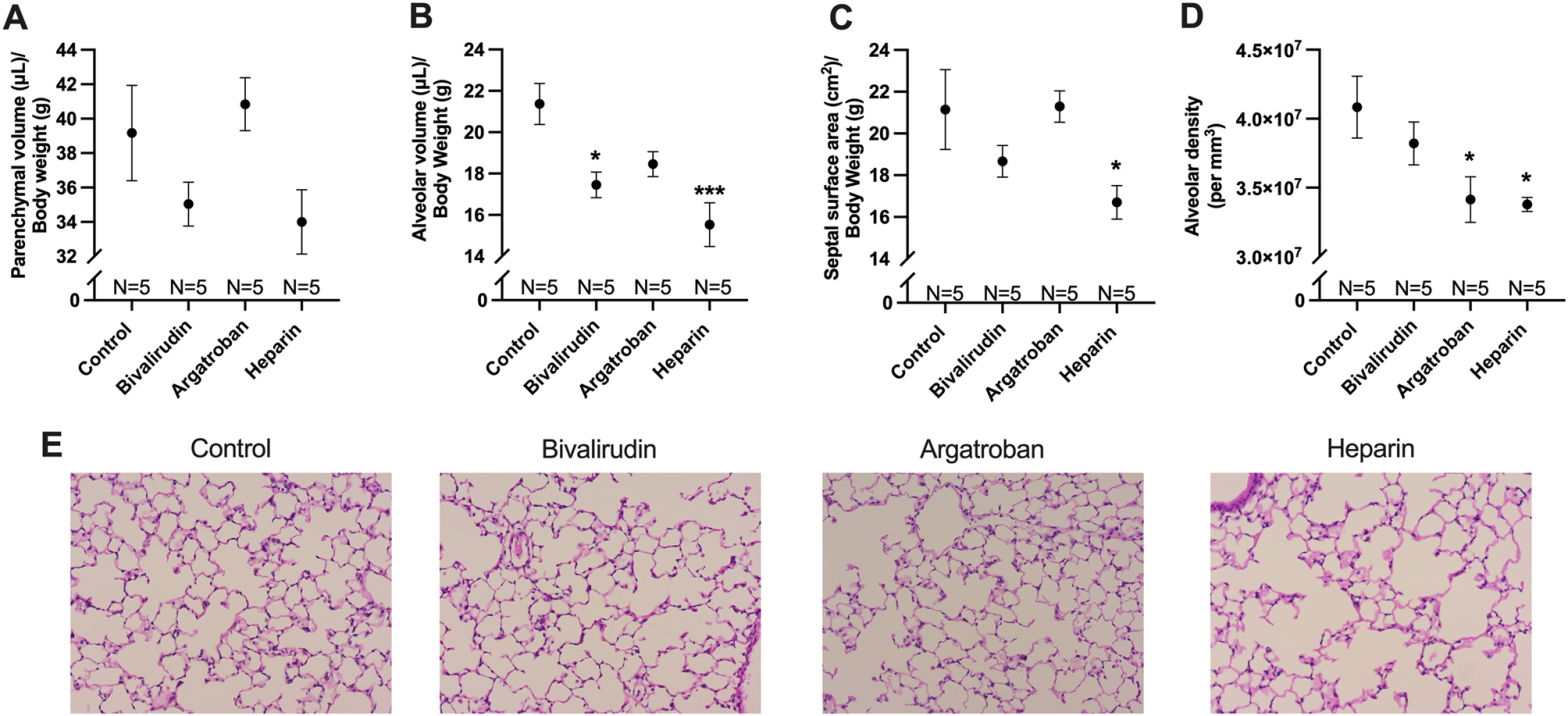
Lung tissue morphometric analysis. Parenchymal volume was lowest for heparin-treated mice compared to the remaining groups, although not reaching statistical significance (**A**). Alveolar volume was significantly lower in the heparin group compared to controls (**B**). Bivalirudin-treated mice also demonstrated lower alveolar volume. Septal surface area, which represents the area available for gas exchange, was significantly lower in heparin mice (**C**). Bivalirudin and argatroban did not significantly impact septal surface area. Alveolar density was significantly lower in the heparin and argatroban groups compared to controls (**D**). Bivalirudin did not significantly change alveolar density. Representative micrographs of hematoxylin and eosin-stained lung sections at 200 × magnification (**E**) demonstrate the decrease in alveolarization in heparin-treated mice compared to the control, bivalirudin, and argatroban groups. Statistical analysis of the experimental groups was done using analysis of variance (ANOVA), with Dunnett’s adjustment for multiple comparisons. Shown are mean ± standard error. *P < 0.05; ***P < 0.001.

Formalin-fixed and paraffin-embedded lungs stained were examined at 200 × magnification for pulmonary morphometric analysis (Fig. 3). There were no significant differences in parenchymal volume across the four groups (Fig. 3A), although heparin-treated mice had the lowest measurement compared to controls (34.0 ± 1.9 vs. 39.2 ± 2.8 μL/g, *P* = 0.19). Alveolar volume (Fig. 3B) was lower in bivalirudin-treated (17.5 ± 0.6 vs. 21.4 ± 1.0 μL/g, *P* = 0.01) and heparin-treated (15.5 ± 1.1 vs. 21.4 ± 1.0 μL/g, *P* = 0.0005) mice. Septal surface area (Fig. 3C), which represents the area available for gas exchange, was lower in the heparin group compared to controls (16.7 vs. 21.2 cm^2^/g, *P* = 0.04). Bivalirudin and argatroban did not significantly change septal surface area (*P* = 0.33, *P* = 1.00, respectively). Finally, alveolar density (Fig. 3D) was significantly lower in the argatroban (3.41 ± 0.17 × 10^7^ vs. 4.08 ± 0.22 × 10^7^ alveoli per mm^3^, *P* = 0.03) and heparin (3.37 ± 0.05 × 10^7^ vs. 4.08 ± 0.22 × 10^7^ alveoli per mm^3^, *P* = 0.02) groups. Bivalirudin-treated mice had similar alveolar density compared to controls (3.82 ± 0.16 × 10^7^ vs. 4.08 ± 0.22 × 10^7^ alveoli per mm^3^, *P* = 0.54).

Representative micrographs obtained at 200 × magnification highlight the preserved alveolarization in the bivalirudin and argatroban, but not heparin groups in comparison to saline-treated controls (Fig. 3E).

### Heparin, but not bivalirudin or argatroban, decreased lung vascularization

**Figure 4:**
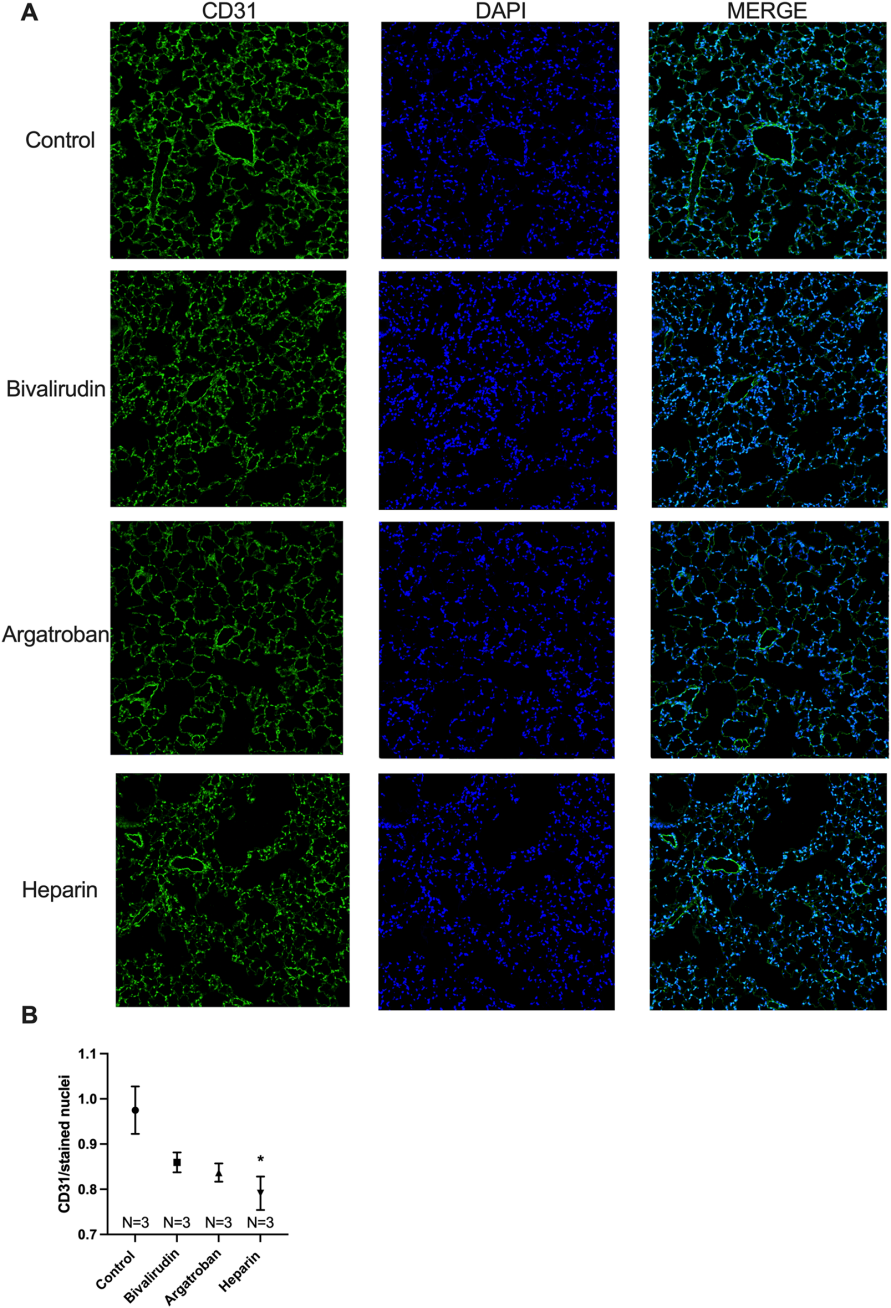
Immunohistochemistry. Representative micrographs at 200 × magnification of co-stained lung tissue for the endothelial cell marker CD31 (green) and nuclear marker DAPI (blue) at postoperative day 8 following left pneumonectomy (**A**). Based on quantification, CD31 was decreased in heparin-treated mice compared to controls (**B**). There was no significant difference in CD31 staining in the bivalirudin or argatroban groups. Statistical analysis of the experimental groups was done using analysis of variance (ANOVA), with Dunnett’s adjustment for multiple comparisons. Shown are mean ± standard error. *P < 0.05.

Lung vascularization was assessed with immunohistochemistry (IHC) by utilizing the CD31 endothelial cell marker, as previously described^16,17^. There were no significant differences in CD31 staining in the bivalirudin (*P* = 0.12) or argatroban (*P* = 0.06) groups although argatroban-treated mice trended towards lower CD31 staining compared to controls (Figs. 4A,B). Heparin treatment resulted in a significant reduction in CD31 staining compared to saline-treated control mice (0.79 vs. 0.97 antibody/stained nuclei, *P* = 0.02).

### Heparin-treated mice had reduced exercise tolerance; bivalirudin and argatroban did not impact exercise performance

**Figure 5:**
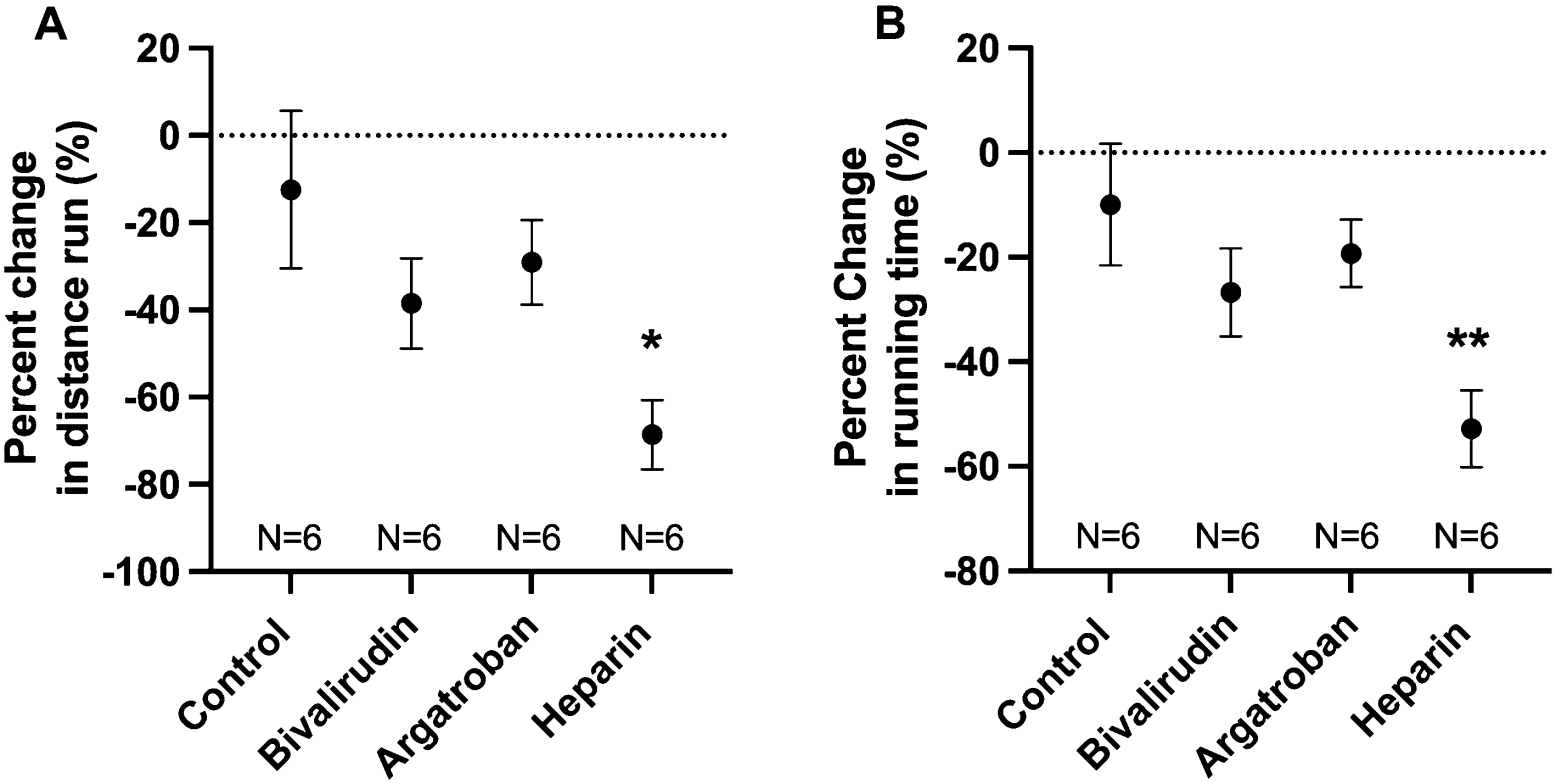
Treadmill exercise tolerance testing (TETT). Mice underwent baseline exercise testing prior to pneumonectomy followed by repeat testing at postoperative day 8. Exercise tolerance was decreased in heparinized mice compared to controls, as measured by the percent change from baseline in distance run (**A**) and time spent running (**B**). There were no significant differences observed in TETT outcomes for the bivalirudin or argatroban groups. Statistical analysis of the experimental groups was done using analysis of variance (ANOVA), with Dunnett’s adjustment for multiple comparisons. Shown are mean ± standard error. *P < 0.05; **P < 0.01.

Mice underwent baseline treadmill exercise tolerance testing (TETT) prior to pneumonectomy and results were compared to repeat testing on POD 8; each mouse thus served as its own control (Fig. 5). All four groups had similar baseline exercise performance assessed by the distance run (*P* = 0.79) and time spent running (*P* = 0.77) (data not shown). Heparin-treated mice had significant percent reduction from baseline in the distance run (Fig. 5A) and running time (Fig. 5B) compared to controls (distance: − 68.6 ± 8.0 vs. − 12.4 ± 18.0%, *P* = 0.02; time: − 52.8 ± 7.3 vs. − 9.9 ± 11.6%, *P* = 0.01). There were no significant differences observed in exercise performance in the bivalirudin (distance: − 38.5 ± 10.3%, *P* = 0.32; time: − 26.7 ± 8.4%, *P* = 0.40) or argatroban (distance: − 29.1 ± 10.3%, *P* = 0.65; time: − 19.3 ± 6.5%, *P* = 0.79) groups.

### Bivalirudin and argatroban, but not heparin, preserved angiogenic signaling

**Figure 6:**
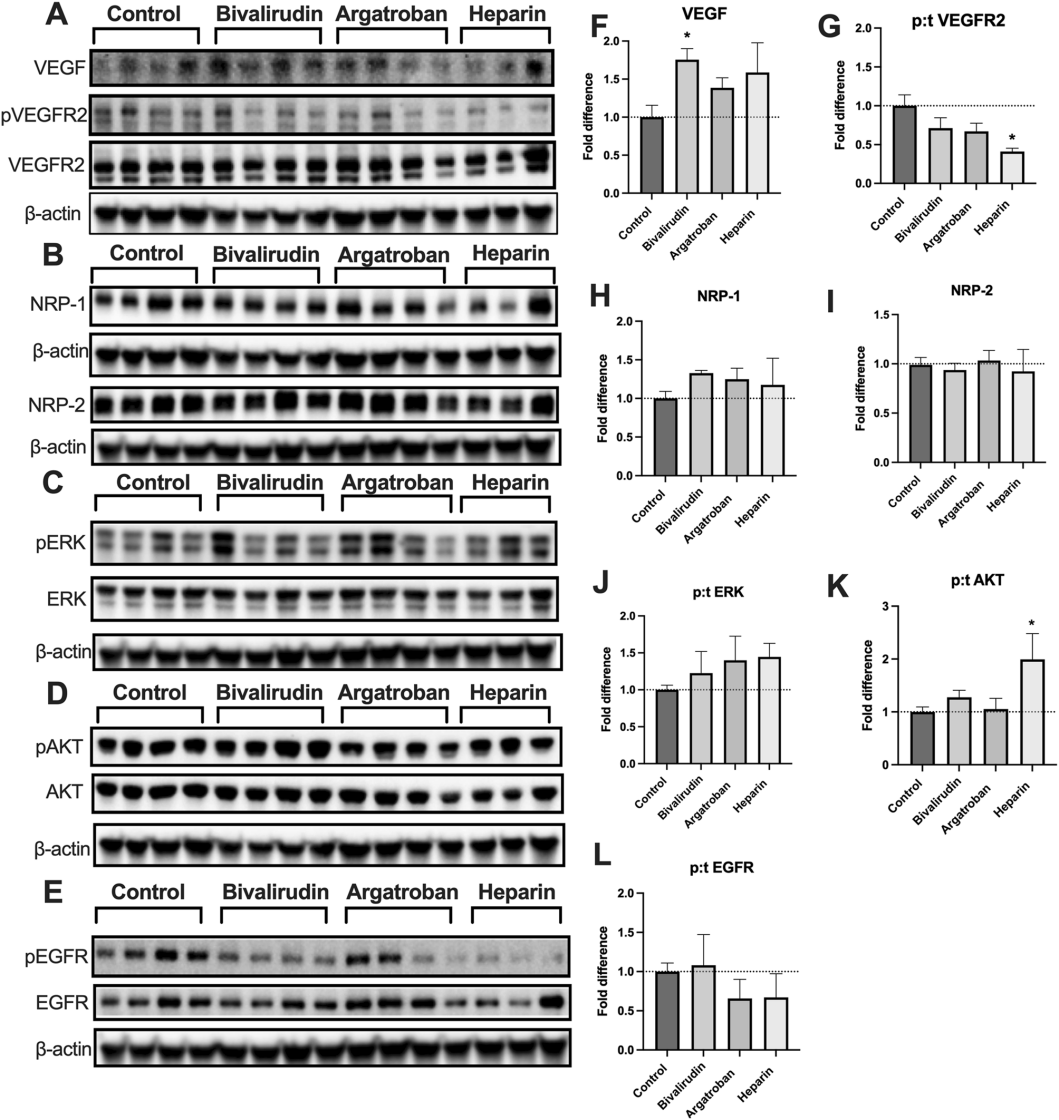
Lung tissue immunoblots. Pulmonary VEGF expression was higher in the bivalirudin group compared to controls (**A,F**) while there were no significant differences in the heparin and argatroban groups. Activation of VEGFR2 (phosphorylated/total receptor VEGFR2) was significantly lower in heparin-treated mice (**G**). There were no significant differences in the expression of the co-receptors neuropilin-1 (NRP-1) or neuropilin-2 (NRP-2) in then bivalirudin, argatroban, or heparin groups (**B,H,I**). Activation of the downstream proliferation marker ERK (phosphorylated/total receptor ERK) was not significantly different in the anticoagulated groups (**C,J**). Heparin-treated mice demonstrated increased activation of the downstream mediator Akt (phosphorylated/total Akt; **D,K**). Activation of an alternative pathway involving the epithelial growth factor receptor (phosphorylated/total EGFR) was lower in the argatroban and heparin groups, although not reaching statistical significance (**E,L**). Uncropped blots are provided in Supplementary Information Files 1,2. The β-actin for each membrane is displayed below the corresponding antibodies and was used to normalize the expression patterns. Each lane represents a sample from a different mouse. Statistical analysis of protein expression was done using analysis of variance (ANOVA), with Dunnett’s adjustment for multiple comparisons. Results are expressed as mean ± standard error. *P < 0.05.

Lung tissue protein homogenates from each group were analyzed via immunoblotting to evaluate for effects of anticoagulation treatment on angiogenic and proliferative signaling pathways (Fig. 6). Endogenous VEGF expression was higher in bivalirudin-treated mice (Fig. 6A,F) compared to controls (*P* = 0.048). There were no significant differences in VEGF expression in the argatroban or heparin groups. Activation of the VEGF receptor (VEGFR2) was decreased 0.41-fold in heparinized mice compared to controls (*P* = 0.02) as demonstrated by the ratio of phosphorylated to total VEGFR2 (Figs. 6A,G). There was no significant difference in VEGFR2 activation for the bivalirudin (*P* = 0.25) and argatroban (*P* = 0.17) groups. Neuropilin-1 (NRP-1) and Neuropilin-2 (NRP-2) are co-receptors for VEGF involved in VEGFR2 signaling^18,19^. There were no significant differences in NRP-1 (Fig. 6B,H) or NRP-2 (Fig. 6B,I) across the four groups.

Despite decreased VEGFR2 activation in heparinized mice, there were no significant differences in the activation of the downstream MAPK/ERK effector pathway in the anticoagulated groups (Fig. 6C,J). However, heparinized mice had increased activation of the downstream mediator Akt (Fig. 6D,K) as demonstrated by the 1.99-fold higher expression of phosphorylated to total Akt compared to controls (*P* = 0.04). There were no significant differences in Akt activation in bivalirudin- or argatroban-treated mice. Activation of the epithelial growth factor receptor (EGFR) was not significantly affected the anticoagulants examined in this study (Fig. 6E,L).

## Discussion

Heparin remains the most used anticoagulant to maintain circuit patency in neonates with CDH or other hypoplastic lung diseases who require cardiopulmonary bypass through ECMO. Our group has previously demonstrated that therapeutic and subtherapeutic heparin impair pulmonary growth and development in the murine CLG model^10,14^. These studies also demonstrated that intermittent bivalirudin dosing did not affect lung growth and function. In our current study, we expand on these previous findings through the use of continuous anticoagulant dosing, by reporting on additional anticoagulants (argatroban), and by presenting additional mechanistic and immunohistochemistry data in the CLG model designed to advance our current understanding.

In an effort to identify suitable alternatives to heparin, we evaluated the effects of the direct thrombin inhibitors bivalirudin and argatroban in vitro on HMVEC-L cells and in vivo utilizing the CLG model. We have demonstrated that, unlike heparin, bivalirudin and argatroban preserved cell proliferation, lung growth, pulmonary alveolarization and vascularization, murine exercise tolerance, and angiogenic signaling.

Initial in vitro studies were first performed on HMVEC-L cells to assess the effects of the anticoagulants on cell proliferation and apoptosis. Microvascular endothelial cells are important in lung growth given prior studies demonstrating their role in pulmonary development and downstream proliferation of alveolar epithelial cells^20–23^. The dose range chosen for each anticoagulant was based on prior reports in the literature to approximate anticoagulation *in vivo*^10,24^. Heparin consistently inhibited HMVEC-L cell proliferation and increased apoptosis (Fig. 1C,F), findings which are consistent with the previously described antiangiogenic effects of heparin^10,25^. Both bivalirudin and argatroban did not affect HMVEC-L proliferation (Fig. 1B,C). Interestingly, bivalirudin had a protective effect by decreasing HMVEC-L cell apoptosis ~ 0.8-fold (Fig. 1E) across all five dosages (3.125–250 μg/mL). The mechanism for the inhibition of endothelial apoptosis requires further investigation, but one recent study suggests that bivalirudin has a protective effect on endothelial cells by blocking thrombin-induced hyperpermeability^26^.

We then utilized subcutaneously implanted osmotic pumps to provide continuous anticoagulation over the course of the eight-day experiment. Dosing was determined by initial dose–response and maintenance studies (Supplementary Fig. 1). The protocol used in the evaluation of anticoagulation function was based on prior studies in mice^27^. Supratherapeutic anticoagulation was utilized in this study to best approximate the clinical scenario of maintaining bypass circuit patency where high levels of anticoagulation are desired^28^. No prior study, to our knowledge, has evaluated the effects of continuous anticoagulation in mice and with the use of osmotic pumps. However, clinically, patients are frequently anticoagulated through continuous dosing and intermittent dosing may not adequately reflect the effects of DTIs which have a shorter half-life compared to heparin (30–45 vs. 60–90 min). An important limitation of our prior work on anticoagulants in the CLG model has been the use of intermittent rather than continuous dosing^10,14^, which does not completely recapitulate the clinical scenario of CDH patients on bypass. Furthermore, while argatroban has been previously used with osmotic pumps^29,30^, these studies did not evaluate anticoagulant activity. Finally, this is the first description regarding the effective delivery of bivalirudin in the induction and maintenance of anticoagulant activity. These results can thus be useful in future anticoagulation studies utilizing the murine model.

Anticoagulated mice underwent lung growth and functional assessment at POD 8 following left pneumonectomy. This time-point was chosen based on prior studies demonstrating CLG completion at that time^9^. Heparinized mice had decreased lung growth compared to controls, while bivalirudin and argatroban did not significantly affect CLG, as evaluated through lung volume measurements (Fig. 2A). Interestingly, there were no significant differences in TLC across the four groups (Fig. 2B). This discrepancy can be explained by considering limitations with the TLC measurement itself. Unlike lung volume, which is measured through water displacement, TLC measurements rely on pressure–volume curves generated following complete alveolar collapse during pulmonary function testing. These measurements can be affected by several uncontrollable variables such as leaks within the system or incomplete degassing. Based on this, and considering prior work^14^, lung volume measurement through water displacement is considered the most reliable and reproducible indicator for lung growth.

Prior studies have demonstrated a correlation between lung growth measured by water displacement and pulmonary morphometric measures such as parenchymal volume and alveolar volume^31,32^. On tissue morphometric analysis, heparin decreased alveolarization as demonstrated by decreased parenchymal volume, alveolar volume, septal surface area, and alveolar density (Fig. 3A–D). Alveolar volume was significantly lower in the bivalirudin group, while argatroban-treated mice had decreased alveolar density. Bivalirudin and argatroban did not impact the remaining morphometric outcomes. To reconcile these findings, it is important to consider alveolarization as a composite of these four morphometric outcomes^32^ and only heparin consistently negatively affected each of these outcomes. Representative micrographs further highlight that bivalirudin and argatroban, unlike heparin, preserved overall alveolarization (Fig. 3E), which correlated with the effect on lung growth. Heparin-treated mice also demonstrated decreased vascularization assessed by staining for the CD31 endothelial cell marker (Fig. 4). This finding is consistent with the previously described link between alveolarization and vascularization during lung development and growth^33^.

The protective effects of DTIs on lung growth and alveolarization correlated with exercise performance (Fig. 5). The use of TETT as a functional measurement of lung growth in murine models is well established^17,31,32^. Heparin resulted in a significant decline in exercise tolerance, as demonstrated by the percent change from baseline in distance run (− 68.6%, *P* = 0.01) and time spent running (− 52.8%, *P* = 0.006). These findings suggest that the negative effects of heparin on lung growth correlate with decreased functional capacity. Importantly, there were no significant differences in TETT outcomes in the bivalirudin or argatroban groups compared to saline-treated controls.

Heparin is known to affect molecular processes implicated in angiogenesis, wound healing, and inflammation, among others^34–36^. At the tissue level, heparin interacts with extracellular matrix proteins and angiogenic growth factors including VEGF^37,38^. Heparin also binds to endothelial angiogenic modulators such as von-Willebrand factor (v-WF) competing with endogenous growth factors^39^. One human prospective cohort study in fact demonstrated that patients treated with heparin had decreased levels of circulating angiogenic peptides compared to those who received bivalirudin^40^.Given this complex array of interactions, it is not surprising that heparin derivatives have been established as potential anti-angiogenic modulators^41^. We hypothesized that bivalirudin and argatroban, given their more specific mechanism of action through direct thrombin inhibition, would not impact angiogenic processes involved in lung growth.

In this study, protein analysis of lung tissue (Fig. 6) confirmed decreased angiogenic signaling in heparinized mice as demonstrated by the 0.41-fold (*P* = 0.02) reduction in the activation of VEGFR2; an important receptor implicated in angiogenesis^42^. Bivalirudin and argatroban did not significantly affect activation of VEGFR2. Interestingly, bivalirudin significantly upregulated upstream expression of VEGF, which considering the inhibition of apoptosis in bivalirudin-treated HMVEC-L cells in vitro, suggests a possible positive effect on lung endothelial cell signaling. The mechanism by which bivalirudin induces these effects warrant further investigation but is beyond the scope of the current study.

Changes in VEGF and VEGFR2 activation did not correlate with changes in the downstream proliferative MAPK/ERK effector pathway (Fig. 6C,J). Furthermore, heparin increased activation of Akt (Fig. 6D,K). One possible explanation for these findings is that alternative pathways involved in lung growth become involved in response to the heparin-mediated inhibition of VEGF signaling. The lung tissue in the heparin group may also reflect an earlier proliferative stage of CLG due to the overall attenuation of lung growth. It is important to consider that protein samples are taken from a single time-point (POD 8) and may fail to fully recapitulate a dynamic continuous process. Regardless, since lung growth is heavily dependent on angiogenic signaling, these findings correlate with the structural and functional outcomes described in this paper.

The use of DTIs in cardiopulmonary bypass is evolving but studies have consistently demonstrated their safety and efficacy^12^. Clinically, bivalirudin was shown to have substantial improvements over heparin in hospital mortality in adult patients on bypass^13^. In fact, many centers are now transitioning infants with CDH and pulmonary hypoplasia to bivalirudin anticoagulation during ECMO. Bivalirudin anticoagulation protocols have demonstrated both safety and efficacy in CDH patients^43^. The findings in this paper demonstrating decreased lung growth in the CLG model with heparin but not bivalirudin/argatroban provide further pre-clinical justification. Clinical studies comparing anticoagulation with DTI compared to heparin on clinical outcomes in CDH are necessary to confirm these findings.

There are several important limitations in this study. CLG is a continuous dynamic process and we have examined a single time-point (POD 8) in the evaluation of functional and structural outcomes. This time-point was selected as CLG is known to be complete based on prior work but this limits interpretation of in vivo signaling pathways^9^. An additional limitation is related to the CLG animal model which has important differences compared to human infants with CDH or pulmonary hypoplasia. While pneumonectomy-induced CLG shares molecular signaling with neonatal lung development^8^, it is not representative of the true anatomic defect in CDH or pulmonary hypoplasia. Furthermore, complex global genetic deficiencies with poorly understood mechanisms are increasingly recognized as important in the pathophysiology of CDH^3^; these processes are not necessarily recapitulated in the CLG model. Within these limitations, the murine CLG model has important similarities to developmental alveolarization as it relates to the formation of new septa, which suggests activation of developmental programming to induce alveolarization^44,45^. Finally, we did not examine the concurrent use of heparin and bivalirudin/argatroban and their effect on lung growth and function in the CLG model. Future investigations should examine this scenario, as even when DTIs are used, patients may still be exposed to heparin for additional clinical indications (e.g. as a line lock solution).

In conclusion, heparin impaired lung growth, alveolarization, vascularization, exercise tolerance, and angiogenic signaling in an established murine model of CLG following left pneumonectomy. Bivalirudin and argatroban did not impact these key outcome measures. These data further support the use of direct thrombin inhibitors for systemic anticoagulation on ECMO in CDH in which expeditious lung growth is essential for survival. Based on this work, clinical studies on the impact of heparin and DTIs on CDH outcomes are warranted.

## Methods

### Cell culture and molecular assays

#### Proliferation and apoptosis assays

Human microvascular lung endothelial cells (HMVEC-L) (Lonza, Morristown, NJ) were plated at 30% confluence in gelatin-coated 96-well plates and starved overnight at 37 °C in basal medium [EGM-2 growth medium + 0.5% fetal bovine serum (Lonza, Morristown, NJ)]. Cells were then washed and treated with increasing concentrations of heparin (0–10 IU/mL), bivalirudin (0–250 μg/mL), or argatroban (0–250 μg/mL), followed by incubation at 37 °C for 72 h. Cell viability was assessed with a Cell Counting Kit-8 (Dojindo Molecular Technologies, Rockville, MD) and apoptosis was then determined using the Caspase-Glo 3/7 assay (Promega, Madison, WI) per the manufacturer’s instructions. The FLUOstar Omega microplate reader (BMG Labtech, Ortenberg, Germany) was used to measure colorimetric and luminescence signals, respectively for each assay.

### Animal experiments

All experiments involving animals were carried out according to the guidelines and regulation of the National Institutes of Health Guide for the Care and Use of Laboratory Animals and the use of Laboratory Animals was approved by the Institutional Animal Care and Use Ethics Committee (Boston Children’s Hospital). This study was carried out in compliance with the ARRIVE guidelines. Eight to ten-week-old C57BL/6 J male mice (Jackson Laboratories, Bar Harbor, ME) weighing approximately 25 g were utilized in this study.

### Assessment of anticoagulant dosing and function

Alzet osmotic pumps (Models 2001/2002, Cupertino, CA) were filled under sterile conditions with normal saline (control), heparin, bivalirudin, or argatroban. Pharmaceutical grade anticoagulants were utilized in this study. For the initial dose–response, different concentrations and/or rates of drug delivery (0.5 μL/hr or 1 μL/hr) were trialed (Supplementary Fig. S1). These values were based on prior reports in the literature^10,29,30^. Pumps were first primed overnight according to the manufacturer’s instructions. The following day, mice were anesthetized with isoflurane (induction: 2–4%, maintenance: 1–3% with 1 L/min oxygen) and received subcutaneous buprenorphine extended-release (3.25 mg/kg; Fidelis Animal Health, North Brunswick, NJ) for pain management. Pumps were then implanted subcutaneously via an incision located midway between the scapulae. Mice were monitored until sternal and ambulatory. Animals were sacrificed 24 h following pump implantation, and blood was collected via inferior vena cava venous puncture in 1:10 dilution of 3.2% trisodium citrate^27^. Plasma was separated by centrifugation (1500 × *g*, 15 min) and coagulation function was assessed through measurement of partial thromboplastin time (PTT)^27^. Coagulation testing was performed by the core hematology laboratory at Boston Children’s Hospital.

The lowest dose of each anticoagulant that resulted in supratherapeutic anticoagulation consistently was then selected for longitudinal studies to ensure systemic anticoagulation throughout the 8-day experiment (Supplementary Fig. S1B–D). Separate cohorts of mice underwent subcutaneous pump implantation as per above and were sacrificed at the following additional time-points: POD 2, 4, and 8. Blood was again collected through inferior vena cava puncture for PTT testing. Based on these results, heparin-treated mice received additional daily intraperitoneal injection of heparin (0.5 IU/g) starting at post-operative day 4 to maintain therapeutic anticoagulation throughout the later time-points of the study. The additional heparin dosing was validated in previous studies from our group^10^. The remaining groups received isovolumetric normal saline (vehicle) intraperitoneal injection for the same frequency/duration.

### Surgical animal model

C57BL/6 J mice were anesthetized with ketamine (80–100 mg/kg) and xylazine (5–10 mg/kg) via intraperitoneal injection. Animals were then intubated under direct visualization with a 22-gauge angiocatheter (Critikon, Tampa, FL) and attached to a rodent ventilator (MiniVent Ventilator; Harvard Apparatus, Holliston, MA) set to deliver 180 breaths/min. A left pneumonectomy was then performed as previously described^7^, followed by subcutaneous placement of Alzet pumps through a mid-scapula incision. Animals were randomized to four experimental groups: normal saline (control), bivalirudin, argatroban, or heparin and received pumps that were pre-loaded with the respective anticoagulant depending on group assignment and dosing plan (Supplementary Table S1). One milliliter of normal saline was subcutaneously administered to provide fluid resuscitation and a single dose of subcutaneous buprenorphine extended-release (3.25 mg/kg; Fidelis Animal Health) was given for postoperative analgesia. Animals were recovered on a heating pad and monitored continuously until sternal and ambulatory. Mice in all experimental groups were sacrificed on POD 8, as CLG is known to be complete at this timepoint^9^. At sacrifice, one cohort of animals underwent pulmonary function testing, lung volume measurement, and lung fixation for staining, while a separate cohort underwent treadmill exercise tolerance testing (TETT) followed by tissue harvest for immunoblot analysis.

### Pulmonary function testing

Mice were deeply anesthetized with ketamine (100 mg/kg) and xylazine (10 mg/kg). The trachea was exposed and a tracheostomy was performed with a 20-gauge hollow needle. Mice were then paralyzed with pancuronium (0.8 mg/kg) and connected to the Flexivent^®^ system (SCIREQ, Montreal, Canada) for PFT measurements as previously described^17^. This system utilizes a forced oscillation maneuver and single compartment model to determine pulmonary compliance (C) and inspiratory capacity. TLC is then measured by ventilating the animal with 100% oxygen for 5 min. After this time, the tracheal tube is completely occluded and the lungs are degassed as the oxygen diffuses into the surrounding tissue and circulation. During this process, the alveoli completely collapse, and the animal is euthanized. TLC is then measured through pressure–volume curves that are generated from three rounds of lung inflation to 35 cm H_2_O followed by deflation to – 10 cm H_2_O. TLC was normalized to mouse body weight.

### Lung volume and morphometric analysis

The remaining right lung was removed and inflated with 10% formalin at 35 cm H_2_O. Lung growth was evaluated by measuring lung volume using the water displacement method^46^ and normalized to body weight. Inflated lung specimens were then formalin-fixed for 24 h at 4 °C and transferred to 70% ethanol. Specimens were paraffin embedded for histologic analysis. Lung sections were stained with hematoxylin and eosin (H&E) for quantitative microscopy (N = 5 per group). In total, 17 lung fields per section were selected at 200 × magnification via systematic uniform random sampling^32^. For each field, a 42-point lattice with grid line was used for quantitative microscopy based on the principles of lung stereology as previously described^47,48^. This technique yields important measurements such as alveolar volume, septal surface area, and mean septal thickness. Volume and area measurements were normalized to mouse body weight. In addition, 10 random sections at 400 × magnification were selected and the number of alveolar units were counted by a masked observer (MMJ).

### Immunohistochemistry

Paraffin-embedded lung sections (N = 3 per group) from each group were assessed for CD31 expression (endothelial cell marker) using immunofluorescence as previously described^17^. Briefly, Histo-clear II (ThermoFisher Scientific, Waltham, MA) was used for deparaffinization followed by progressive rehydration with decreasing concentrations of ethanol and then water. Epitope retrieval was achieved by incubating in a citrate-based unmasking solution (Vector Laboratories, Burlingame, CA) at 120 °C in a pressurized chamber (Decloaking Chamber, Biocare Medical, Pacheco, CA). Sections were permeabilized with 0.05% Tween-20 in phosphate-buffered saline (PBST) for 30 min, and blocked in 5% donkey serum (Abcam, Cambridge, UK) for 1 h. Slides were incubated overnight with anti-CD31 (Cell Signaling Technology, Danvers, MA) at 4 °C. Following overnight incubation, slides were washed with phosphate buffered saline (PBS) and PBST and incubated with Alexa-Fluor-conjugated donkey anti-rabbit secondary antibody (ThermoFisher Scientific, Waltham, MA) for 2 h at room temperature. Sections were counterstained and mounted using Fluoroshield™ with DAPI (Sigma-Aldrich, St. Louis, MO). Specimens were examined using confocal microscopy (LSM 880, Zeiss, Jena, Germany) at 200 × magnification and random 25-tiled high-power fields (HPF) spanning the entire lung were captured for analysis. ImageJ v1.53a (National Institutes of Health, Bethesda, MD) was used to quantify signal intensity.

### Treadmill exercise tolerance testing

A separate cohort of mice in each group underwent treadmill exercise tolerance testing. TETT was used as a metric of functional outcome following pneumonectomy as previously described^32^. Mice were individually placed on a stationary treadmill with an attached shock grid (Exer 3/6 Treadmill, Columbus Instruments, Columbus, OH) and acclimated to the apparatus for five minutes at 6 m/min. After acclimation, the velocity was set to increase at a rate of 2 m/min. Mice were exercised until exhaustion, defined as remaining on the shock grid for more than 5 s.

TETT was performed two days prior to pneumonectomy for baseline measurement, and again at POD 8. Distance run and time spent running were compared in each mouse pre- and post- pneumonectomy, and reported as a percent change compared to baseline. This was done in order to account for behavioral and intrinsic physiologic differences between mice. Following TETT, the exercised mice were euthanized. At sacrifice, blood was collected via inferior vena cava venous puncture in 1:10 dilution of 3.2% trisodium citrate. Plasma was separated by centrifugation (1500 × *g*, 15 min) and anti-factor Xa level was measured by the core hematology laboratory at Boston Children’s Hospital to ensure that additional heparin dosing resulted in maintained anticoagulation. At sacrifice, the remaining right lung was also harvested for molecular analyses as described below.

### Western immunoblot

Approximately 40 μg of lung tissue from each group was suspended in Tissue Protein Extraction Reagent with added protease and phosphatase inhibitors (T-PER™; ThermoFisher Scientific, Waltham, MA). Samples were homogenized and centrifuged for 10 min at 12,000 rpm (13,523 × *g*) and 4 °C. The supernatant was collected and the protein concentration was determined using the Bradford assay (BioRad, Hercules, CA). Protein samples were suspended in 1 × Laemmli buffer (Boston Bioproducts, Mildford, MA) and separated on an SDS 4–10% PAGE gel. Samples were then transferred to polyvinyl difluoride (PVDF) membranes (Merck Millipore, Darmstadt, Germany). Membranes were blocked in 5% nonfat milk in tris-buffered saline with Tween 20 (TBST) for one hour and incubated in 1:500 to 1:1000 dilution of primary antibodies in 5% nonfat milk-TBST at 4 °C overnight. Primary antibodies included anti-p-Y1175-VEGFR2, -VEGFR2, -NRP1, -NRP2, -pT202/Y204-ERK, -ERK, -p-S473-AKT, AKT, -p-Y1068-EGFR, -EGFR (Cell Signaling Technology, Danvers, MA), and anti-VEGF_120/164_ (R&D Systems, Minneapolis, MN). After washing with TBST, membranes were incubated with horseradish peroxidase (HRP)-conjugated secondary (anti-rabbit or anti-goat) antibody (R&D Systems, Minneapolis, MN) for one hour at room temperature (1:2000 dilution). Blots were normalized by probing with HRP-conjugated β-actin antibody (Sigma-Aldrich, St. Louis, MO). Immunoblots were developed using enhanced chemiluminescence reagents (Bio-Rad, Hercules, CA) on a ChemiDoc Touch System Imager (Bio-Rad, Hercules, CA). Signals were quantified with Image Lab Software v6.1.0 (Bio-Rad, Hercules, CA).

### Statistical analysis

Comparison across the four groups for the various outcomes in this study was done using a one-way analysis of variance (ANOVA) with Dunnett’s adjustment for multiple comparisons. The normal saline group served as the control group in all analyses. Results are expressed as mean ± standard error (SE). The SE was selected in this study to describe the variation of the population of samples, an approach justified by the prior work of Festing and Altman^49^. For all analyses, *P* < 0.05 was considered statistically significant. Statistical analyses were performed on GraphPad Prism v9 (La Jolla, CA) and confirmed by our biostatistician (PDM).

## Supplementary Information

Supplementary Information.

## Data Availability

The datasets generated and/or analyzed during the current study are available from the corresponding author (MP) on reasonable request.

## Abbreviations

CD31: Cluster of differentiation 31
CDH: Congenital diaphragmatic hernia
CLG: Compensatory lung growth
DTI: Direct thrombin inhibitors
ECMO: Extracorporeal membrane oxygenation
EDTA: Ethylenediaminetetraacetic acid
EGFR: Epidermal growth factor receptor
ELISA: Enzyme-linked immunosorbent assay
HPF: High power field
HRP: Horseradish peroxidase
H&E: Hematoxylin and eosin
HMVEC-L: Human microvascular endothelial cells-lung
IHC: Immunohistochemistry
MAPK/ERK: Mitogen-activated protein kinase/extracellular signal-regulated kinase
NRP-2: Neuropilin-2
PBS: Phosphate-buffered saline
PBST: Phosphate-buffered saline with 0.05% Tween-20
PFT: Pulmonary function testing
PVDF: Polyvinyl difluoride
pEGFR: Phosphorylated epithelial growth factor receptor
pVEGFR2: Phosphorylated vascular endothelial growth factor 2
PTT: Partial thromboplastin time
RIPA: Radioimmunoprecipitation assay
TBST: Tris-buffered saline with Tween 20
TETT: Treadmill exercise tolerance testing
TLC: Total lung capacity
VEGF: Vascular endothelial growth factor
