# Supplementary material for: Direct thrombin inhibitors as alternatives to heparin to preserve lung growth and function in a murine model of compensatory lung growth
Source: Sci Rep. 2022 Dec 7;12:21117. doi: 10.1038/s41598-022-25773-3 (PMC9729628; doi:10.1038/s41598-022-25773-3)

**Direct Thrombin Inhibitors as Alternatives to Heparin to Preserve Lung Growth and Function in a Murine Model of Compensatory Lung Growth**

Savas T. Tsikis[1,2], Thomas I. Hirsch[1,2], Scott C. Fligor[1,2], Amy Pan[1,2], Malachi M. Joiner[1,2], Angela Devietro[1,2], Paul D. Mitchell[3], Hiroko Kishikawa[1,2], Kathleen M. Gura[4], Mark Puder*[1,2]

[1]Vascular Biology Program, Boston Children's Hospital, Harvard Medical School, Boston, MA 02115, USA
[2]Department of Surgery, Boston Children's Hospital, Harvard Medical School, 300 Longwood Ave, Fegan 3, Boston, MA 02115, USA
[3]Institutional Centers for Clinical and Translational Research, Boston Children's Hospital, Boston, MA 02115, USA
[4]Department of Pharmacy and the Division of Gastroenterology and Nutrition, Boston Children's Hospital, Harvard Medical School, Boston, MA, USA.

***Corresponding Author:**
Mark Puder, MD PhD
Boston Children's Hospital
Department of Surgery
300 Longwood Ave, Fegan 3
Boston, MA, 02115
Phone: 617-355-1838
Fax: 617-730-0477
Mark.Puder@childrens.harvard.edu

**Supplementary Table, Figure Legends and Files**

**Supplemental Table S1.** Study groups and anticoagulant final dosing

| GROUPS | CONCENTRATION | PUMP RATE | DELIVERY |
|---|---|---|---|
| **Normal Saline (Control)** | N/A | 1 μL/hr | **1 μL/hr** |
| **Bivalirudin** | 50 μg/μL | 1 μL/hr | **50 μg/hr** |
| **Argatroban** | 100 μg/μL | 0.5 μL/hr | **50 μg/hr** |
| **Heparin** | 2.5 IU/μL | 1 μL/hr | **2.5 IU/hr*** |

N/A: Not applicable; IU: International units
* Heparin-treated mice received an additional daily intraperitoneal injection of heparin (0.5 IU/g) starting at post-operative day 4. Remaining groups received daily intraperitoneal injection of isovolumetric saline vehicle.

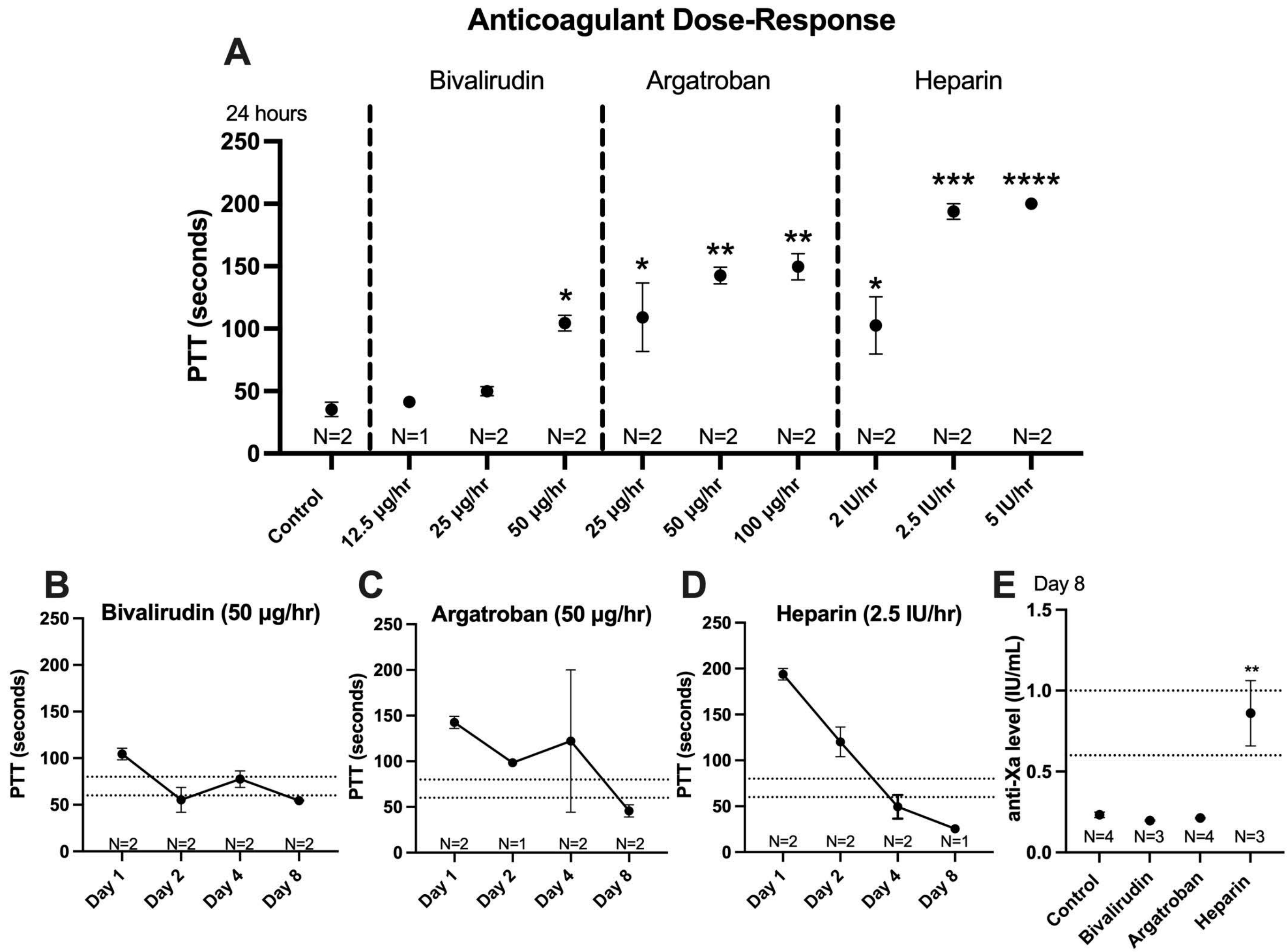


**Supplemental Figure S1. Anticoagulation dose-response and maintenance.** Different concentrations and rates of drug delivery were trialed during initial dose-response studies for each anticoagulant. Increasing the rate of drug delivery increases the level of anticoagulation at 24 hours after pump implantation (**A**) as measured by the partial thromboplastin time (PTT). The lowest dose of each anticoagulant was then selected to ensure maintenance of anticoagulation throughout the 8-day study. Both bivalirudin- (**B**) and argatroban-treated (**C**) mice maintained elevated PTTs as measured at various time-points. Heparin-treated mice were noted to have decreasing PTTs reaching comparable levels to controls starting on Day 4 (**D**). These mice received additional daily intraperitoneal injection of heparin (0.5 IU/g) to maintain therapeutic anticoagulation throughout the study. Heparin significantly increased anti-factor Xa level compared to controls at day 8 after left pneumonectomy (**E**). Comparison were done using a one-way analysis of variance (ANOVA) with Dunnett's adjustment for multiple comparisons. Shown are mean ± standard error. Normal PTT range: 25.0-37.0; Normal anti-factor Xa range: 0.30-0.70 are indicated on the graph. **P*<0.05; ***P*<0.01; ****P*<0.001; *****P*<0.0001.

**Supplemental File 1**. Full length blots correspond to part of Figures 5A, 5B, 5D:

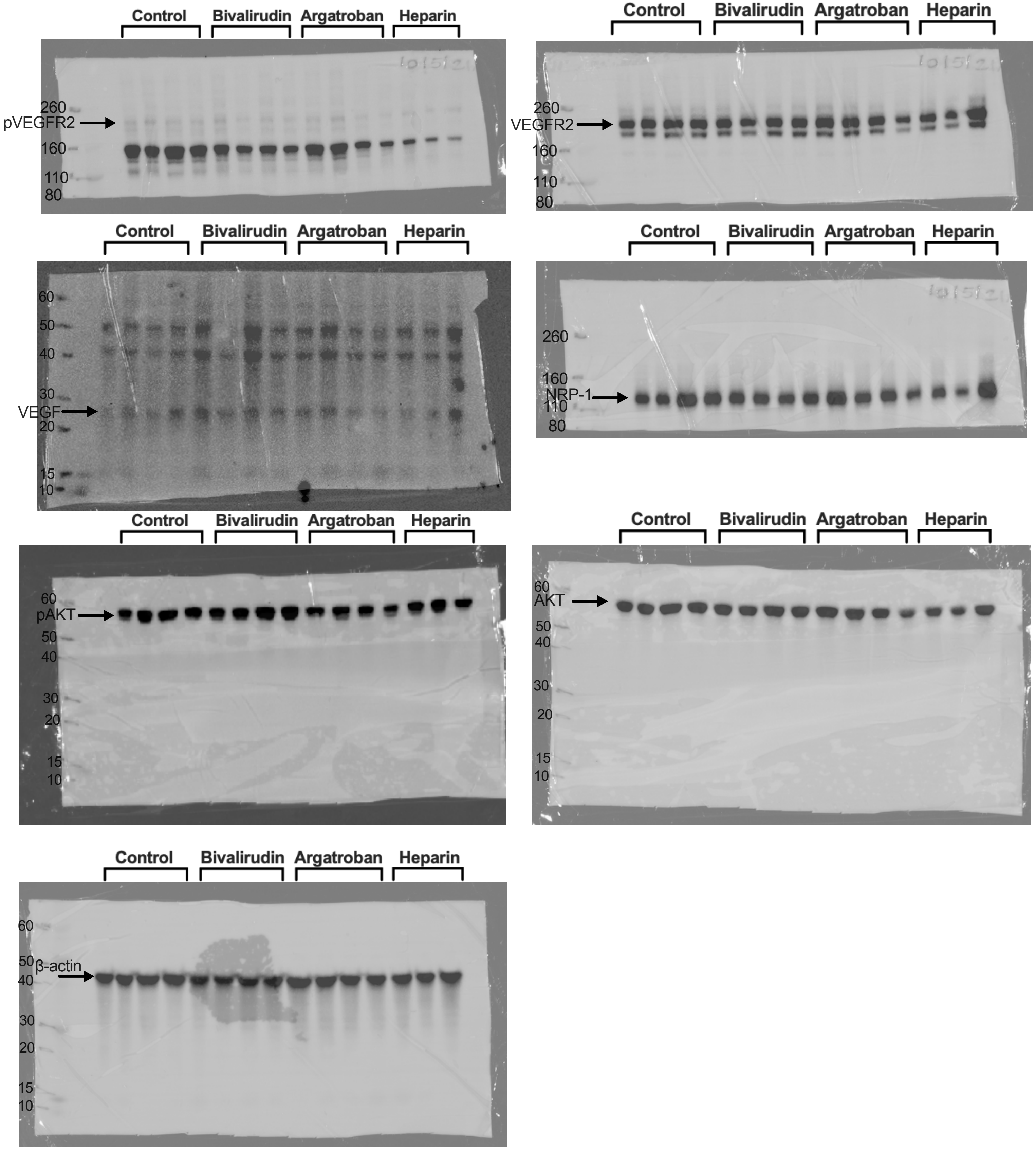

**Supplemental File 2**. Full length blots correspond to part of Figures 5B, 5C, 5E:

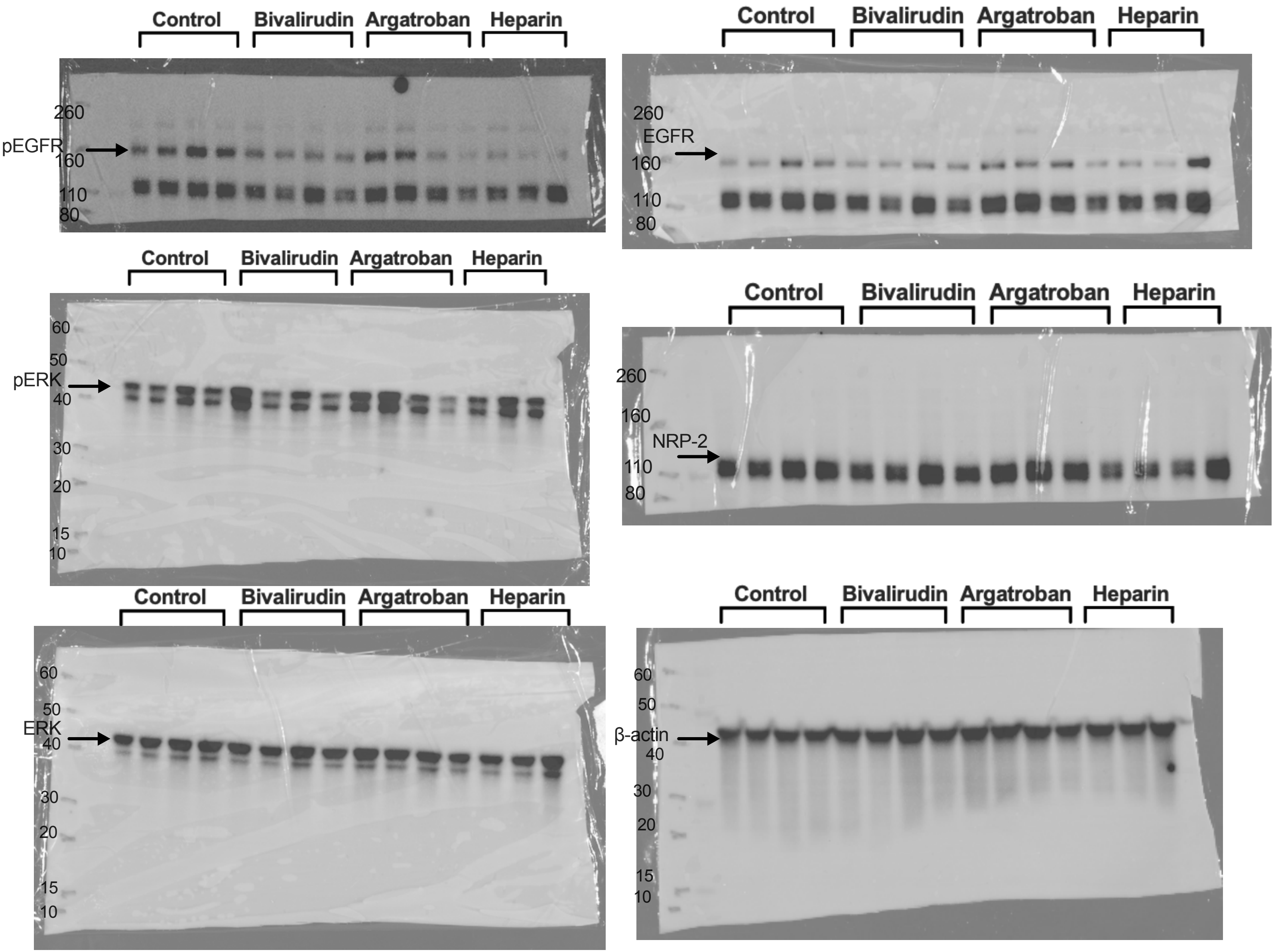

Supplement: Supplementary file 1 — Supplementary Information. [file 41598_2022_25773_MOESM1_ESM.pdf]
